# Supplementary material for: Preoperative nutritional evaluation of patients with hepatic alveolar echinococcosis
Source: PLoS One. 2020 Feb 24;15(2):e0229396. doi: 10.1371/journal.pone.0229396 (PMC7039506; doi:10.1371/journal.pone.0229396)
Supplement: S1 File — (DOC) [file pone.0229396.s002.doc]

**
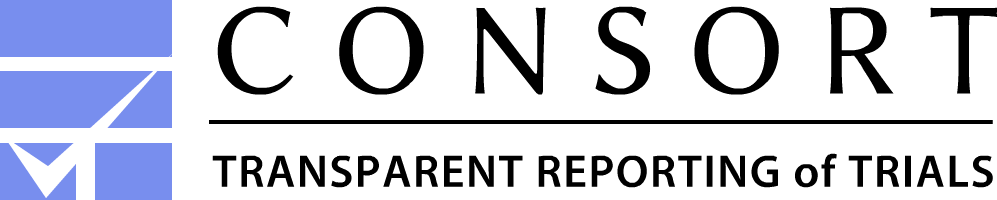
**

**CONSORT 2010 Flow Diagram**

**Allocation**

**Analysis**

**Follow-Up**

**Enrollment**

Assessed for eligibility (n= 93)

Excluded (n= 239 )

♦  Not meeting inclusion criteria (n=238 )

♦  Declined to participate (n= 1)

♦  Other reasons (n=0 )

Analysed (n=93 )
♦ Excluded from analysis (give reasons) (n= 0 )

Lost to follow-up (give reasons) (n= 93 )

Discontinued intervention (give reasons) (n= 0)

Allocated to intervention (n= 93 )

♦ Received allocated intervention (n= 93 )

♦ Did not receive allocated intervention (give reasons) (n=0 )

Lost to follow-up (give reasons) (n=0 )

Discontinued intervention (give reasons) (n=0 )

Allocated to intervention (n=0 )

♦ Received allocated intervention (n=0 )

♦ Did not receive allocated intervention (give reasons) (n=0 )

Analysed (n= 0)
♦ Excluded from analysis (give reasons) (n=0 )

Randomized (n= 93 )
