# Supplementary material for: Preoperative nutritional evaluation of patients with hepatic alveolar echinococcosis
Source: PLoS One. 2020 Feb 24;15(2):e0229396. doi: 10.1371/journal.pone.0229396 (PMC7039506; doi:10.1371/journal.pone.0229396)
Supplement: Attachment — Submitted filename: Response to Michelle Ellis.doc [file pone.0229396.s006.doc]

**Dear Michelle Ellis,**

We thank you for reviewing our manuscript entitled "Preoperative nutritional evaluation of patients with hepatic alveolar echinococcosis" (ID：PONE-D-19-21935). Your comments have considerably helped to revise and improve our paper, in addition to providing important guidance to our research. We have studied the comments carefully and made corrections which we hope meet your approval. The main corrections are incorporated in the manuscript and responses to the reviewers’ comments are provided below.

**Replies to the Journal Requirements**

1. Thank you for including your ethics statement on the online submission form:

   'This study has been approved by the Medical Ethics Committee of the Affiliated
   Hospital of North Sichuan Medical College.
   All patients have been informed suitably, and we've asked for their verbal consent.
   We've obtained consent from parents or guardians when our study included minors
   under age 18.'

   To help ensure that the wording of your manuscript is suitable for publication, would you please also add this statement at the beginning of the Methods section of your manuscript file.

**Response：**We have added this statement at the beginning of the Methods section of our manuscript files (‘Manuscript.doc’ and ‘Revised Manuscript with Track Changes.doc’). Thank you.

2.

1) Please clarify if the data present in your supporting information file, "S1 Table" qualifies as the minimal data set underlying your study. Please note, PLOS defines the “minimal data set” to consist of the data set used to reach the conclusions drawn in the manuscript with related metadata and methods, and any additional data required to replicate the reported study findings in their entirety.

   If the minimal data set underlying your study is present in the file "S1 Table," we would like to propose the following data availability statement for your approval:

   "All relevant data are in the paper and Supporting Information files"

   Can you please confirm the above statement is both complete and correct? With your approval, we can update your data availability statement on your behalf.

   2) If "S1 Table" is not the minimal data set underlying your study, please clarify in detail how future researchers can access all of the minimal data set. Please provide any relevant information, such as DOIs, URLs, or accession codes a future researcher may need.

   With the information your provide, we can update your data availability statement on your behalf.

**Response：**Thank you for pointing this out. We sure that the minimal data set underlying our study is present in the file "S1 Table" and all relevant data are in the paper and Supporting Information files. You can update our data availability statement on our behalf, thanks.

**Once again, we appreciate your constructive comments and suggestions which have helped to improve the clarity and depth of the paper.**

**Yours sincerely,**

**Xie Liang**

**Email address:** [**scuxl@foxmail.com**](mailto:scuxl@foxmail.com)

**Corresponding author: Xu Mingqing**

**Email address:** [**xumingqing@scu.edu.cn**](mailto:xumingqing@scu.edu.cn)

**Email address:** [**scuxl@foxmail.com**](mailto:scuxl@foxmail.com)

**Corresponding author : Xu Mingqing**

**Email address:** [**xumingqing@scu.edu.cn**](mailto:xumingqing@scu.edu.cn)
